# Natural product library screening identifies Darutigenol for the treatment of myocardial infarction and ischemia/reperfusion injury

**DOI:** 10.1186/s13020-025-01141-x

**Published:** 2025-06-18

**Authors:** Kun Liu, Li Zheng, Qian-Yu Huang, Hong-Ji Li, Cheng Li, Hui Zhao, Ze-Bing Ye, Hao Wang, Xu-Feng Qi, Meng Wang

**Affiliations:** 1 Department of Cardiology, Zhongshan Torch Development Zone People’s Hospital, Zhongshan, 528437 China; 2 Key Laboratory of Regenerative Medicine of Ministry of Education, Institute of Aging and Regenerative Medicine, Department of Developmental & Regenerative Biology, and Department of Cardiology, The Affiliated Guangdong Second Provincial General Hospital, Jinan University, Guangzhou, 510632 China; 3 School of Environmental Science and Engineering, Guangdong University of Technology, Guangzhou, 510006 China; 4 Key Laboratory of Regenerative Medicine of Ministry of Education, School of Biomedical Sciences, Faculty of Medicine, The Chinese University of Hong Kong, Shatin, Hong Kong SAR China; 5 Department of Cardiology, The Affiliated Guangdong Second Provincial General Hospital, Jinan University, Guangzhou, 510632 China; 6 Department of Anesthesiology & Clinical Research Center, The First Affiliated Hospital, Jinan University, Guangzhou, 510632 China

**Keywords:** Drug screening, Darutigenol, Myocardial infarction, Ischemic/reperfusion injury

## Abstract

**Introduction:**

Ischemic heart diseases are the leading cause of death worldwide due to the inability of regeneration of adult cardiomyocytes (CMs). Natural products from medical herbs are an important source of innovative drugs for many diseases including cardiovascular diseases.

**Objectives:**

In this study, we set out to screen novel small-molecule therapies from natural products to protect heart against ischemic injury.

**Methods:**

High-throughput screening was performed using a natural product library to identify the potential small molecules which can promote survival of CMs under ischemic and ischemic/reperfusion conditions. In addition, myocardial infarction (MI) and ischemia/reperfusion (I/R) mice models were used to evaluate the in vivo effects of the screened candidate. We also applied various analysis including cell viability, qPCR, Western blot, immunofluorescent staining, echocardiography, Masson’s staining, TTC staining, and network pharmacology.

**Results:**

High-throughput screening showed that the small molecule compound Darutigenol (Dar), derived from the Chinese traditional herb *Herba Siegesbeckiae*, could significantly promote CM survival and proliferation under ischemic conditions. Moreover, I/R-induced CM apoptosis and ROS generation could be significantly reduced by Dar treatment. In addition, in vivo administration of Dar was able to attenuate MI- and I/R-induced cardiac injury in adult mice by decreasing fibrosis and apoptosis, thereby improving cardiac function. Network pharmacology analysis and molecule docking assay showed that Dar has the highest binding affinity with AKT1 protein. Western blotting assay further revealed that AKT1 activation was significantly enhanced by Dar administration in the infarcted hearts.

**Conclusions:**

Our data revealed that the small molecule compound Dar, screened from the natural product library in this study, is capable of protecting heart against MI and I/R injury by activating AKT1 pathway. These findings enrich the natural product candidates for cardiovascular disease treatment and provide new insights into potential therapeutic agents for MI and I/R injury.

## Introduction

Ischemic cardiovascular disease, the leading cause of morbidity and mortality, produces tremendous health and economic problems around the world [1]. Acute myocardial infarction (MI) caused by coronary occlusion is the largest contributor to cardiac dysfunction and heart failure. Due to mammalian cardiomyocytes possessing very limited self-renewal ability after birth, injured heart after MI unable to replenish cardiomyocytes (CMs) to repair itself [2, 3]. The infarcted area will be replaced with nonfunctional collagen and fibrotic scar. Timely resupply of coronary perfusion using the therapeutic methods such as thrombolytic therapy and percutaneous coronary intervention is vital to reduce cardiomyocytes death [4]. However, during the course of restoration of blood flow into the ischemia heart, it also induces immense CMs injury termed as ischemia/reperfusion (I/R) injury [5]. Therefore, investigating management options to induce resident CM division and proliferation as well as fibrosis resolution is a promising strategy for heart repair. As small molecule drugs have plenty of advantages, including reduced cost and immune response, and easily to find pharmacologic target for drugs. Based on natural compound library screening, the molecules bufalin and lycorine have been identified as drug candidates for therapeutic applications in cardiac fibrosis and diastolic dysfunction [6]. In a latest study, artesunate was identified as an efficient agent for cardiac fibrosis treatment from a library of ∼5,000 compounds using a high-throughput screening [7]. These previous reports suggest that the natural small molecule compounds may be a promising resource to identify therapeutic agents for the treatment of cardiovascular disease.

In this study, we performed a high-throughput screening using a natural product library of 1,271 compounds, and found that the small molecule compound Darutigenol could efficiently protect CMs in the in vitro and in vivo models of MI and I/R injury. Darutigenol (Dar) is a natural product derived from the aerial parts of the traditional Chinese herb *Herba Sigesbeckia* (HS). HS belongs to the family of Asteraceae that is widely distributed in many countries in Asia. Many traditional Chinese medicine prescriptions containing HS have been used to treat diseases, such as rheumatoid arthritis, hypertension, snakebites and malaria [8, 9]. Researchers have demonstrated that the extracts of HS possess the efficacy to mitigate inflammatory and algetic response [10], to attenuate cartilage degradation and bone erosion in mouse rheumatoid arthritis model [11], to suppress tumor cells growth in vitro [12], to alleviate cardiac I/R injury in rats [13]. However, as a main ingredient of HS, the potential roles of Dar in cardiac MI and I/R injury remain largely unknown. In the present study, we demonstrated that Dar administration can promote survival of CMs under MI and I/R conditions through the activation of AKT1 pathway, thereby attenuating myocardial MI and I/R injury.

## Materials and methods

### High-throughput drug screening

Briefly, HL-1 cells (a mouse cardiac muscle cell line) were cultivated in 96-well plates at a density of 2,000 cells per well in a high-glucose DMEM medium containing 0.2% FBS and 1% penicillin–streptomycin. 24 h after seeding, the cells within the in positive control group were cultured with 10% FBS, while the drug treatment and negative control groups were respectively subjected to a library of 1,271 natural products (Selleck, L1400) at a final concentration of 10 μM and vehicle at the same concentration, respectively. Cells were then treated for 48 h at 37 °C in a 5% CO_2_ humidified incubator and subsequently incubated with 10 μL CCK-8 solution (Beyotime, C0039) for another 1 h before being loaded onto the micro plate reader (Biotec) to detect cell viability. The “proliferation index” was determined by the following equation: proliferation index = OD_450nm_ in drug treatment group/OD_450nm_ in negative group.

### Animals

Neonatal and adult C57BL/6 J mice (male, 8–10 weeks) were purchased from Guangdong Zhiyuan Biopharmaceutical Technology Co., Ltd (Guangzhou, China). Mice were feed in a specific pathogen-free (SPF) laboratory animal facility of Jinan University (Guangzhou, China). The animal experiments described in this study were conducted according to the Guidelines on the Care and Use of Laboratory Animals for biomedical research, as published by the National Institutes of Health (No.85-23, revised 1996). All animal protocols and procedures were approved by the Laboratory Animal Committee of Jinan University (IACUC-20210607-06).

### Isolation of neonatal mice cardiomyocytes

The hearts of P3 mice were collected and washed two times with PBS (Servicebio, G0002) to remove the remaining blood. Then, the hearts were cut into small chunks of ~ 1 mm3 and transferred to a 50 mL centrifuge tube containing 10 mL of digestive buffer I (0.0125% trypsin–EDTA; Gibco, 25200056 and 10.11 mg/mL BDM; Sigma, B0753). The tube was then subjected to constant stirring at 4 °C for a duration of 12 h. Subsequently, the supernatant was discarded and 10 mL of digestive buffer II containing collagenase II (1.5 mg/mL; Gibco, 17101015) and BDM (2 mg/mL) was added. The mixture was stirred continuously in a 37 °C water bath for 15 min and slowly blown 10 times with a pipette. After digestion, cells were collected by filtration through a 70 μm cell strainer. The filtrate was centrifuged at 660 rpm for 5 min, after which the cell precipitate was resuspended in 5 mL of high-glucose DMEM containing 10% FBS and 1% penicillin–streptomycin and then seeded onto 6-cm cell dishes for 2 h at 37 °C in a 5% CO_2_ humidified incubator. Suspended cardiomyocytes were then collected and seeded onto cell culture plates pre-treated with Lamin (14.3 μg/mL; Gibco, 354232). After 24–48 h, isolated cardiomyocytes were used to corresponding experiments [14].

### Immunofluorescence staining

To assess the effect of candidate molecules on CM proliferation, the cell proliferative phase marker Ki67, and phosphorylated histone H3 (pH3) were utilized. For Ki67 and pH3 staining, CMs were rinsed three times with PBS and fixed with 4% paraformaldehyde (Servicebio, G1101) for one hour after stimulation with candidate agents for two days. Primal antibodies of Ki67 (Servicebio, GB111499, 1:500) and pH3 (CST, 9701S, 1:300) were then co-incubated with cTnT antibody (Proteintech, 15513-1-AP, 1:300) to the CMs, respectively, following blocking with 5% BSA for one hour at room temperature. Next, CMs were rinsed three times with PBS and incubated with nucleic dye DAPI (Beyotime, C1006) for 15 min. Finally, the CMs were incubated with CoraLite488-conjugated Goat Anti-Mouse IgG (Proteintech, SA00013-1, 1:300) and Cy3-conjugated Goat Anti-Rabbit IgG (Proteintech, SA00009-2, 1:300) for one hour at room temperature and then washed three times with PBS. For each sample, at least 10 images were captured at random. More than 2,000 CMs were counted per sample.

### Mouse myocardial infarction (MI) injury model

MI injury in adult C57BL/6 J mice (8–10 weeks) was induced by permanent ligation of the left anterior descending artery (LAD) as previously described [15]. Briefly, mice were anesthetized by intraperitoneal injection of 250 μL of pentobarbital sodium (70 mg/mL, Sigma-Aldridge, P3761). The hairs on the throat and chest of mice were removed and sterilized with 75% ethanol and povidone iodine solution. After connecting the mice's trachea to the ventilator, the chests of the mice were opened between the third and fourth ribs on the left side of the mice to expose the heart. Using an 8–0 silk suture to permanent ligation of the LAD to induce MI, the cavity in the chest and throat were sutured with a 6–0 silk suture. For sham operated mice, all the steps are the same as above, except that the LAD is not ligated.

### Mouse myocardial ischemia/reperfusion (I/R) injury model

I/R injury in adult mice was established by short-term ligation of the left anterior descending artery (LAD) as previously described [16]. Operations like anesthesia, endotracheal Intubation, and thoracotomy were the same as those described in the MI model above. However, in the step of ligation LAD, a 2–3 mm long piece of PE-10 tubing needed to be placed over the LAD. The tubing and LAD were then ligated using a 7–0 silk suture, inducing myocardial ischemia. Following a 45-min period, the tubing over the LAD was then removed in order to re-perfuse the blood to the heart. In the sham-operated mice, all the aforementioned steps were replicated, with the exception of LAD ligation. Finally, the cavity in the chest and throat were sutured with a 6–0 silk suture.

### Echocardiography

The cardiac function of the mice was measured using echocardiography (VINNO 6 LAB, China) at specific time points following the induction of MI. Left ventricle systolic function, including left ventricle ejection fraction (EF, %) and fractional shortening (FS, %), was determined by measuring M-mode echocardiography from the short axis of the left ventricle at the papillary muscle level.

### Masson trichrome staining

Mice hearts were embedded in paraffin and cut in 5 μm-thick sections, and stained with Masson's Trichrome staining Kit (Solarbio, G1340) in accordance with manufacturers'manuals. Heart sections were scan using digital microscope (Motic Easyscan 6, China). The normal areas of the heart are stained red by Ponceau-Acid Fuchsin solution, while areas that are infarcted are stained blue by Aniline Blue solution. The infarcted area of cardiac sections was measured by ImageJ software.

### Wheat germ agglutinin (WGA) staining

Paraffin-embedded heart sections were stained with WGA solution (WGA, 5 μg/mL, Thermo Fisher, W11261) in darkness for 15 min at room temperature. After washed three times by PBS, the sections were photographed by confocal microscopy (Olympus FV3000, Japan). To measure the cross-section area of CMs, more than 10 fields and 200 cells were analyzed for each heart section using ImageJ software.

### Quantitative real-time PCR (qPCR)

Total RNA was extracted from mice hearts using RNA isolater Total RNA Extraction Reagent (Vazyme, R401-01). 1 μg total RNA was reverse transcription to cDNA using HiScript II Q Select RT SuperMix (Vazyme, R233-01). The qPCR was performed using ChamQ Blue Universal SYBR qPCR Master Mix (Vazyme, Q312-02) on the MiniOpticon qPCR System (Bio-Rad, USA). GAPDH was used as the internal standard control to normalize gene expression using the 2^−ΔΔCt^ method. The sequences of the qPCR primers are as follows: α-SMA-F, 5’-AGG GAG TAA TGG TTG GAA TGG-3’; α-SMA-R, 5’-GGT GAT GAT GCC GTG TTC TA-3’; Col-3-F, 5’-AGC CAC CTT GGT CAG TCC TA-3’; Col-3-R, 5’-GTG TAG AAG GCT GTG GGC AT-3’; GAPDH-F, 5’-TGT GTC CGT CGT GGA TCT GA-3’; GAPDH-R, 5’-CCT GCT TCA CCA CCT TCT TGA-3’.

### Western blotting

Total protein was isolated from mice hearts using RIPA lysate buffer (Beyotime, P0013B) supplied with a 1 × protease inhibitor cocktail (Beyotime, P1005) at 4 ℃ for 30 min. Lysates were centrifuged for 10 min at 14,000 × g and supernatants were collected as whole cell extracts. Protein concentration was quantified using a BCA protein quantification test kit (Servicebio, G2026). 50 μg total protein per sample was separated on 12% SDS-PAGE gels and transferred to polyvinylidene difluoride membranes. Membranes were blocked with 5% BSA and incubated with the indicated primary antibodies. Primal antibodies used in this study are following: anti-Bax (CST, 14796, 1:1000), anti-Bcl2 (Huabio, ET1702-53, 1:1000), anti-GAPDH (Proteintech, 60004-1-Ig, 1:10000). Secondary antibodies used are following: goat-anti-mouse horseradish peroxidase (HRP)-conjugated antibody (CST, #7076, 1:5000) and goat-anti-rabbit horseradish peroxidase (HRP)-conjugated antibody (CST, #7074, 1:5000). Signals were detected with the ChemiDoc XRS chemiluminescent gel imaging system (Analytik Jena). The relative expression levels of the target protein were analyzed using ImageJ software.

### TTC staining

The hearts of mice were harvested and washed twice in Phosphate Buffered Saline (PBS) after 24 h of cardiac I/R injury. The hearts were then flash frozen at −80 ℃ for 10 min. After this, the hearts were sliced into 1 mm-thick sections and stained with 1% 2,3,5-triphenyl tetrazolium chloride (TTC) solution (Servicebio, G1017) in the dark for 15 min at 37 ℃. The area at the ischemic zone displayed a white color due to the absence of dehydrogenase enzymes, while the healthy area presented a red color. The heart sections were photographed by digital camera (Canon, Japan). The infarcted area of the heart sections was then measured using ImageJ software.

### TUNEL staining

To evaluate the effect of candidate molecules on CMs apoptosis after oxygen–glucose deprivation/reperfusion (6 h/18 h) insult, TUNEL staining assay was used in accordance with manufacturers'manuals (Servicebio, G1502).

### Network pharmacology analyze

The molecular structure of the natural product Darutigenol (Dar) was obtained from the PubChem database. The SwissTargetPrediction and PharmMapper databases were utilized to predict the potential target of Dar. The ischemic heart disease-related targets were obtained from five databases, including DisGeNET, Genecards, TTD, and CTD. The predicted Dar targets were then overlapped with the ischemic heart disease-related targets to obtain the intersection targets by the Venny map. The intersection targets were subsequently imported into the String database to construct the network of drug-target-disease. The key targets of Dar were then screened by topology analysis in Cytoscape software [17]. The David database was used to analyze the function of the molecule and the signaling pathway involved. Furthermore, the AutoDock Vina software was used to predict the binding affinity between Dar and its targets. The docking results were visualized by PyMOL software [18].

### Statistics

The statistical analysis was conducted using GraphPad Prism 8 Software. For experiments involving three or more groups, statistical analysis was performed using one-way or two-way ANOVA with Dunnett's multiple comparisons post hoc tests. Comparisons between two groups were analyzed using unpaired and 2-tailed Student's t-test. All data are presented as the mean ± SEM. A *p* value of less than 0.05 was considered to be statistically significant.

## Result

### Chemical screening for small molecules that protect cardiomyocytes against ischemic injury

**Fig. 1:**
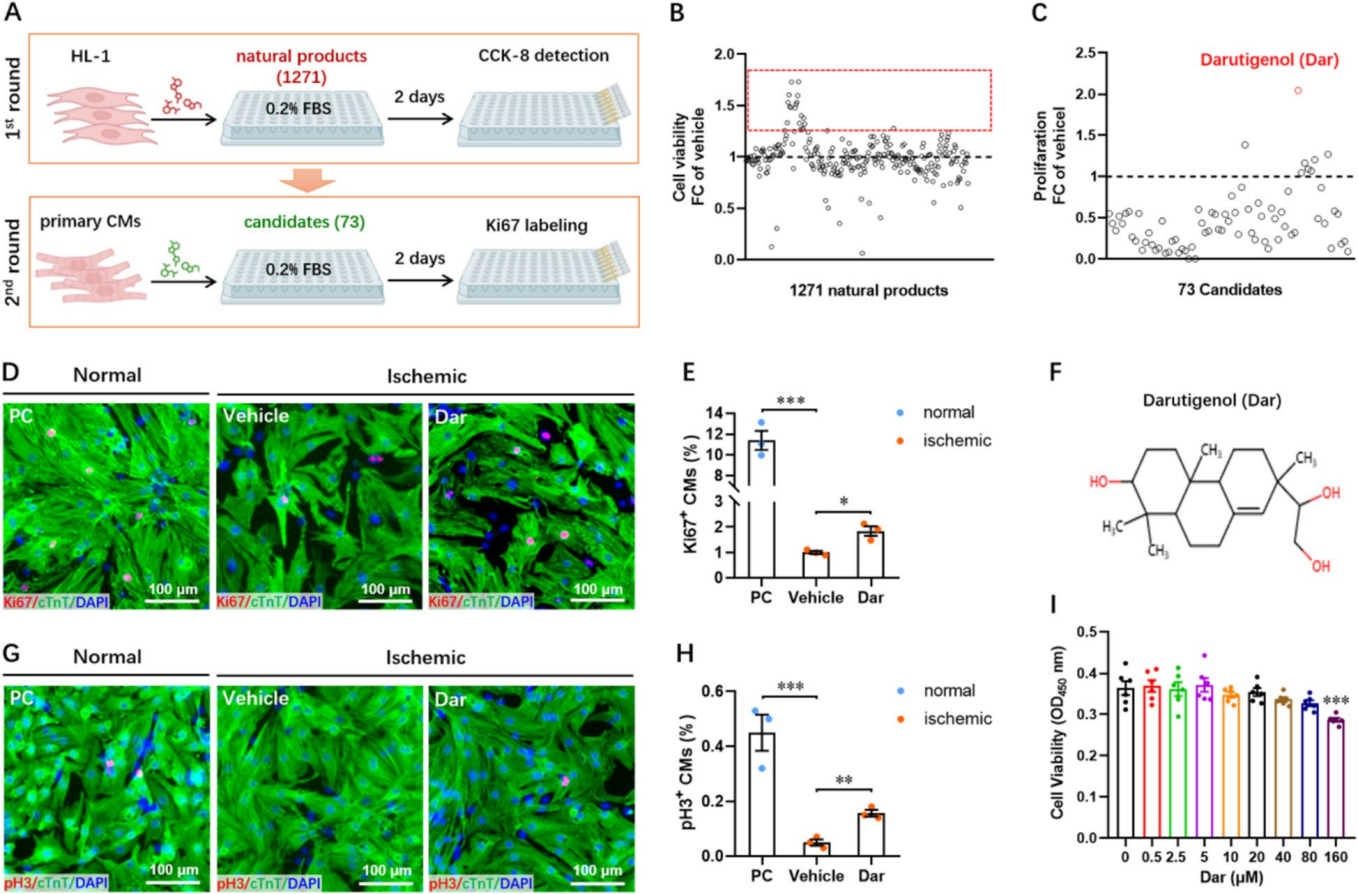
Dar screening for CM survival under ischemic conditions. **A** Schematic of screening of natural products promoting CMs survival and proliferation under ischemic conditions. **B** First screening of 1,271 natural compounds using cell viability assay in HL-1 cells under ischemic conditions. Seventy-three candidates promoting cell survival fold change (FC) ≥ 1.3-fold are selected for the second screening. **C** Second screening of 73 candidates using Ki67 staining in neonatal primary CMs under ischemic conditions. One candidate (Darutigenol, Dar) promoting CM proliferation fold change (FC) ≥ twofold is highlighted by the red circle. **D** and **E** Representative images (**D**) and quantification (**E**) of Ki67^+^ CMs under normal and ischemic conditions with or without Dar treatment (10 μM for 48 h) (*n* = 3). **F** The 2D chemical structure of Dar. **G** and **H** Representative images (**G**) and quantification (**H**) of pH3^+^ CMs under normal and ischemic conditions with or without Dar treatment (10 μM for 48 h) (*n* = 3). **I** The effect of different concentrations of Dar (from 0.5 to 160 μM) on the cell viability of neonatal primary CMs. All data are presented as the mean ± SEM of three separate experiments. *p < 0.05, **p < 0.01, ***p < 0.001 versus control

To screen the potential natural products protecting cardiomyocytes against ischemic injury, a natural monomer product library was used to treat HL-1 cells (a mouse cardiac muscle cell line) under ischemic conditions (0.2% FBS). Firstly, we performed a high-throughput screening based on cell counting assay to identify compounds that promote HL-1 cells survival under ischemic conditions (0.2% FBS). The 10% FBS was used as a positive control (PC). The first round of screening showed that 73 out of 1,271 compounds led to an at least 1.3-fold increase in cell viability under ischemic conditions compared with vehicle group (Fig. 1A, B). Next, a second round of screening was performed to evaluate whether these 73 candidate molecules could improve the proliferation of primary cardiomyocytes (CMs) isolated from neonatal mouse under the ischemic conditions. Among these 73 potential candidates, Ki67 staining revealed that only one molecule named Darutigenol (Dar), an extract of the Chinese traditional herb *Herba Siegesbeckiae*, significantly increased CMs proliferation by 2-folds at a concentration of 10 μM when compared with the vehicle group (Fig. 1C–F). To prove complete cardiomyocyte proliferation, the late cell cycle marker phospho-Histone H3 (pH3) was further used in this study. Our data showed that ischemia treatment greatly decreased the percentage of pH3^+^ CMs compared with positive control group. However, Dar treatment significantly increased the percentage of pH3^+^ CMs under ischemic conditions (Figure G and H). These findings suggest that Dar treatment really promotes the proliferation of cardiomyocytes under ischemic conditions. To evaluate the potential cytotoxicity of Dar, primary CMs were treated with different concentrations of Dar, ranging from 0.5 to 160 μM. The result showed that Dar had no apparent cytotoxicity to primary CMs even at a dose of 80 Μm (Fig. 1I). Collectively, these data indicate that Dar treatment could significantly protect primary CMs from ischemic injury in vitro.

### Dar protects CMs from ischemia/reperfusion (I/R) injury in vitro

**Fig. 2:**
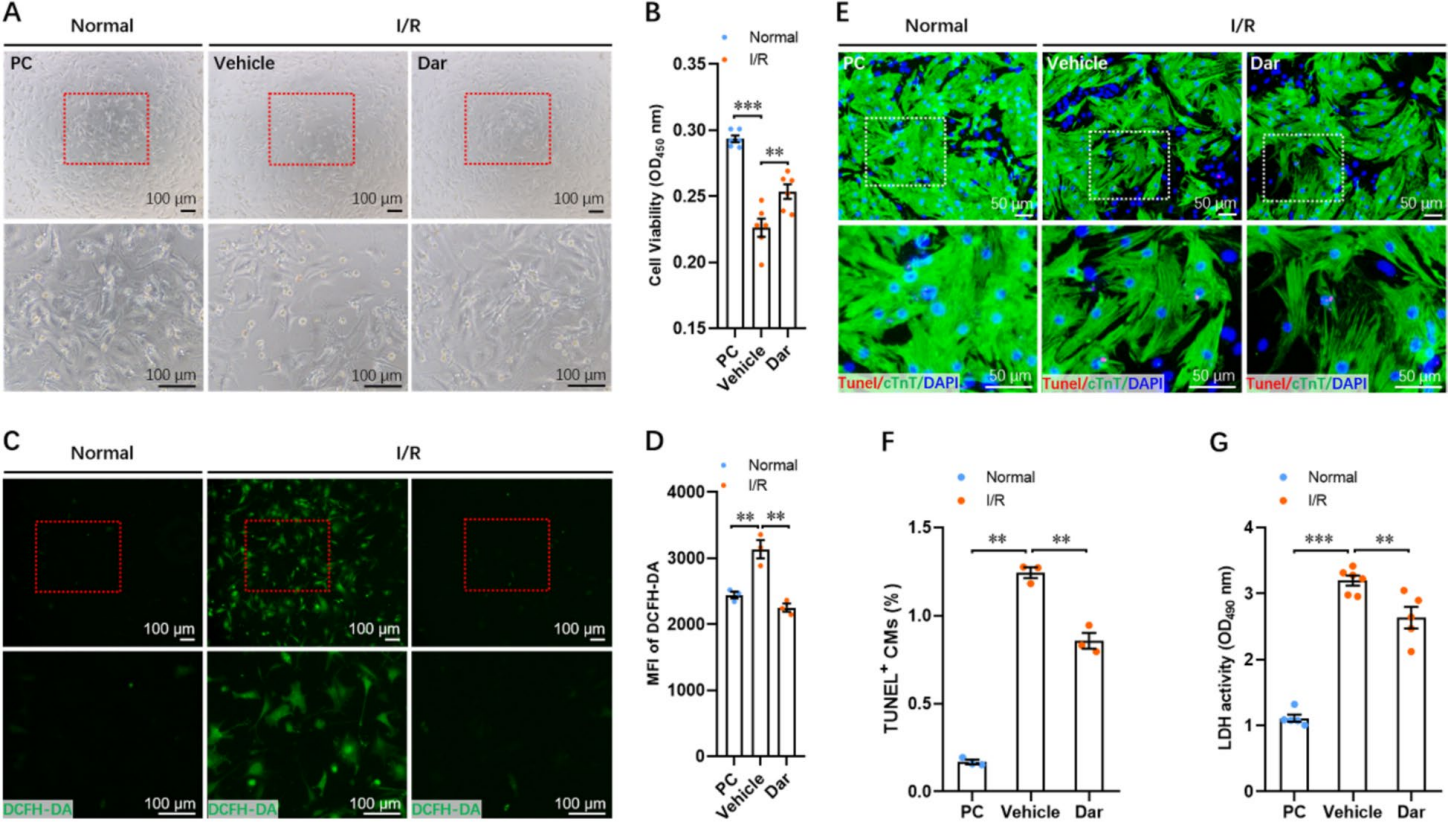
Dar protects primary CMs from I/R injury in vitro. **A** and **B** Representative morphology (**A**) and cell viability quantification (**B**) of neonatal primary CMs treated with or without Dar (10 μM) under I/R conditions (*n* = 3). **C** and **D** Representative images (**C**) and quantification (**D**) of DCFH-DA staining of CMs treated with or without Dar (10 μM) under I/R conditions (*n* = 3). **E** and **F** Representative images (**E**) and quantification (**F**) of TUNEL^+^ CMs treated with or without Dar (10 μM) under I/R conditions (*n* = 3). **G** The LDH activity in supernatants of CMs was detected under different conditions (*n* = 3). All data are presented as the mean ± SEM of three separate experiments. **p < 0.01, ***p < 0.001 versus control

To further determine whether Dar treatment protects CMs from ischemia/reperfusion (I/R) injury in vitro, primary CMs isolated from neonatal mice at p1 were subjected to hypoxia/re-oxygenation (H/R) injury, an ideal in vitro model of myocardium I/R injury. We found that the cell viability of CMs was greatly reduced by I/R, whereas Dar treatment significantly elevated the cell viability of CMs under the condition of I/R (Fig. 2A, B), indicating the protective effects of Dar treatment on cardiomyocytes upon I/R injury. DCFH-DA staining showed that intracellular ROS generation in primary CMs was greatly induced by I/R. However, the increased ROS generation could be significantly reduced by Dar treatment (Fig. 2C, D), implying the antioxidant function of Dar in CMs upon I/R injury. In consistent with this observation, TUNEL staining revealed the anti-apoptotic function of Dar in primary CMs under I/R conditions, as demonstrated by the decreased percentage of TUNEL^+^ CMs in Dar-treated group compared with vehicle-treated group (Fig. 2E, F). In line with TUNEL staining, I/R injury remarkably increased LDH release in primary CMs, whereas the I/R-induced LDH release was significantly blocked by Dar treatment (Fig. 1G). These findings suggest that Dar protects primary CMs from I/R injury by reducing ROS generation, cellular apoptosis, and LDH release.

### Dar improves cardiac dysfunction induced by myocardial infarction

**Fig. 3:**
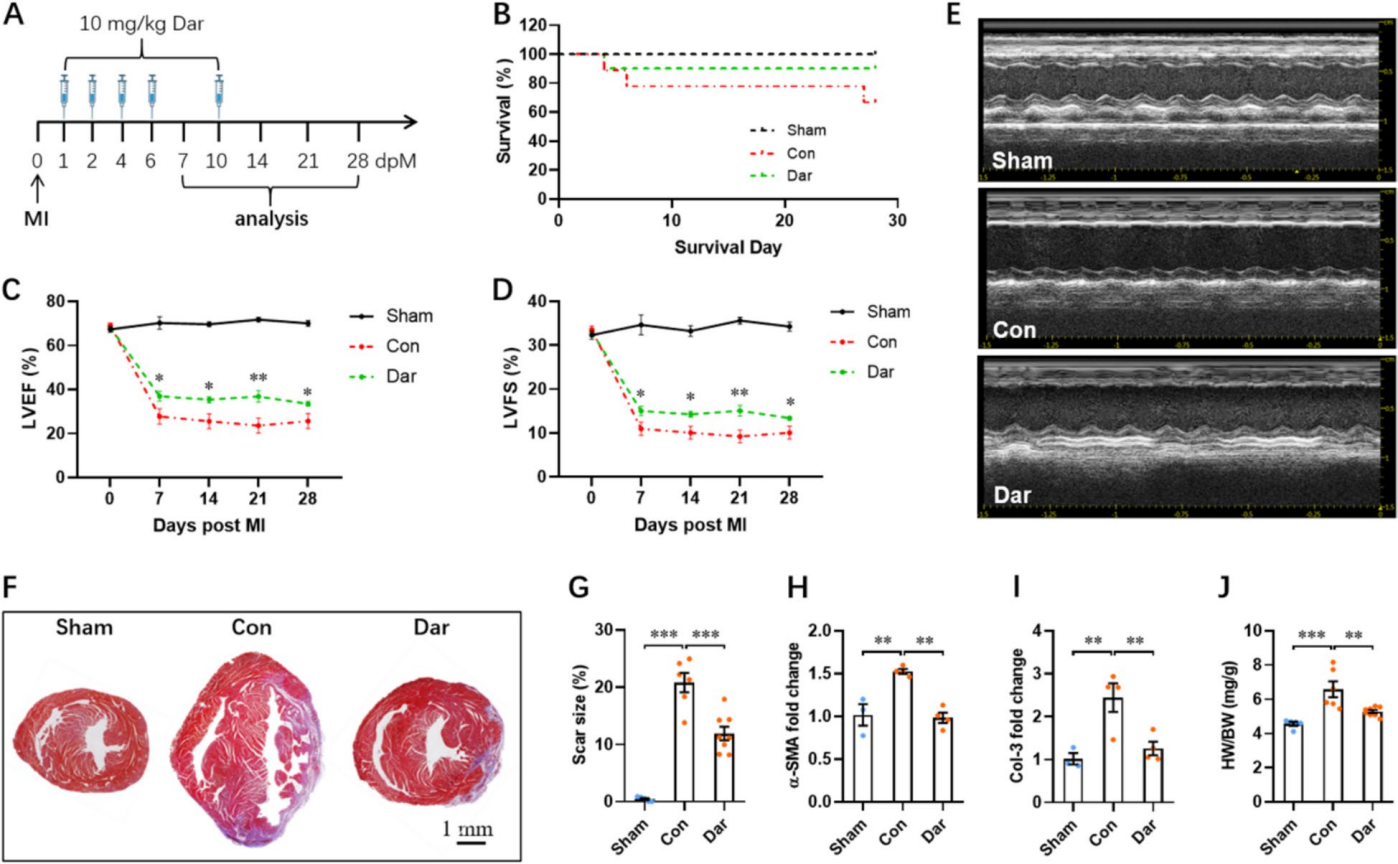
Dar improves cardiac dysfunction induced by myocardial infarction (MI). **A** Schematic of MI injury and Dar administration in adult mice. The mice received MI injury and vehicle injection were used as control group. The mice only received vehicle injection but not MI injury were used as sham group. **B** Survival analysis of mice in sham, control, and Dar-treated groups (*n* = 5 ~ 9). **C** and **D** Quantification of LVEF (**C**) and LVFS (**D**) levels in control (vehicle) and Dar-treated mice from 0 to 28 dpM (*n* = 5 ~ 9 mice). **E** Representative images of M-model echocardiography in sham, control, and Dar-treated mice at 28 days post-MI (dpM). **F** and **G** Representative masson’s trichrome staining images (**F**) and quantification of scar size (**G**) in sham, control, and Dar-treated mice at 28 dpM (*n* = 5 ~ 8 mice). **H** and **I** The qPCR validation of fibrosis markers including α-SMA (**H**) and Col-3 (**I**) in sham, control, and Dar-treated hearts at 28 dpM (*n* = 3 ~ 4 mice). **J** The HW/BW ratio in sham, control, and Dar-treated groups at 28 dpM (*n* = 5 ~ 8 mice). All data are presented as the mean ± SEM of three separate experiments. *p < 0.05, **p < 0.01, ***p < 0.001 versus control

To explore whether Dar treatment improves cardiac dysfunction induced by myocardial ischemia injury, adult male mice were subjected to myocardial infarction (MI) injury, followed by intraperitoneally injection of Dar (10 mg/kg) for five times within 10 days (Fig. 3A). The sham and control groups were administered the same volume of vehicle (Fig. 3A). Survival analysis indicated that there were no significant differences between the sham, control, and Dar-treated groups within 30 days post-MI (dpM) (Fig. 3B). To determine whether Dar treatment influences cardiac function, the echocardiographic analysis was performed from 0 to 28 dpM. Our data showed that cardiac function was improved in Dar-treated mice compared with control group from 7 to 28 dpM, as demonstrated by the increased levels of ejection fraction (EF) and fractional shortening (FS) (Fig. 3C–E). Consistently, Masson’s staining revealed that Dar treatment significantly reduced the scar size at 28 dpM compared with control group (Fig. 3F, G), implying a reduced fibrosis in Dar-treated hearts. In agreement with this result, MI injury remarkably increased the expression of fibrotic markers including α-SMA and Col-3 compared with sham group, whereas Dar treatment significantly suppressed the expression of α-SMA and Col-3 compared with control group (Fig. 3H, I). Histological analysis showed that the increased heart weight to body weight ratio (HW/BW) in control group was significantly reduced by Dar treatment (Fig. 3J).

**Fig. 4:**
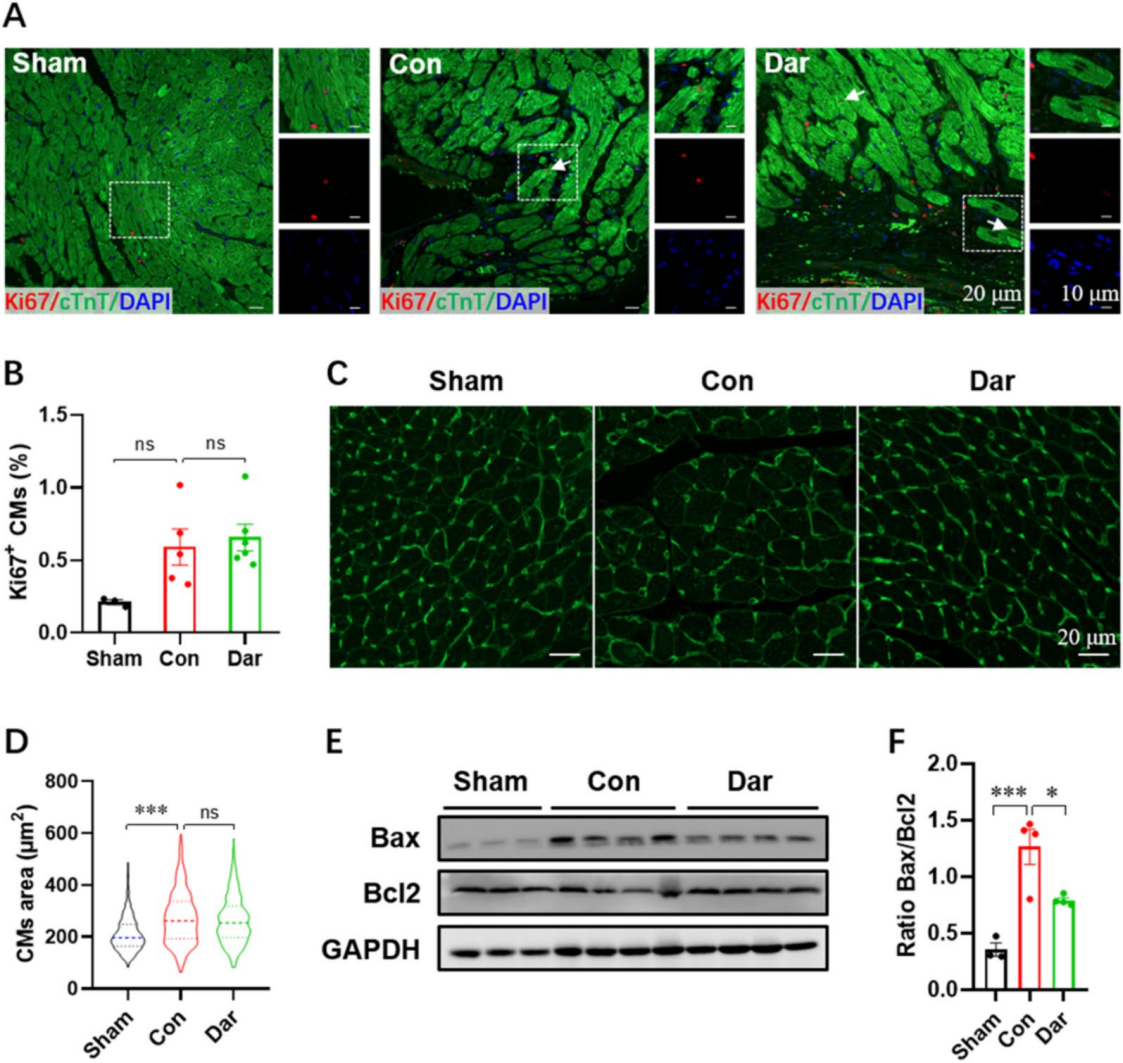
Effects of Dar treatment on CM proliferation, hypertrophy, and apoptosis in the infarcted hearts. **A** and **B** Representative images (**A**) and quantification (**B**) of Ki67^+^ CMs in the border zone of injured hearts at 28 dpM (*n* = 3 ~ 6 hearts). At least 1000 CMs near the injury in each heart were counted. **C** and **D** Representative WGA staining images (**C**) and quantification (**D**) of the size of CMs located in ventricles at 28 dpM (total ~ 500 CMs in 4 hearts per group). **E** and **F** Representative images (**E**) and quantification (**F**) of western blotting for Bax and Bcl2 expression in hearts at 28 dpM (*n* = 3 ~ 4 hearts per group). All data are presented as the mean ± SEM of three separate experiments. *p < 0.05, ***p < 0.001 versus control. Ns denotes there was no significant difference

To further determine whether Dar facilitates heart repair after MI by inducing the proliferation of CMs, we performed Ki67 staining and found that there were no significant differences in Ki67^+^ CMs between control and Dar-treated groups (Fig. 4A, B). The WGA staining revealed that MI injury greatly increased CM size compared with sham group, whereas Dar administration has no significant impacts on the increased CM size induced by MI injury (Fig. 4C, D). Subsequently, we evaluated the effect of Dar on CMs apoptosis in the context of MI. As evidenced by a western blot assay, Bax/Bcl2 ratio in hearts was greatly increased by MI injury, which was further significantly suppressed by Dar administration (Fig. 4E, F), exhibiting an anti-apoptotic effect of Dar against MI injury. Taken together, these data suggest that Dar treatment might attenuate MI injury by decreasing cardiac fibrosis and apoptosis.

### Dar attenuates I/R-induced cardiac injury

**Fig. 5:**
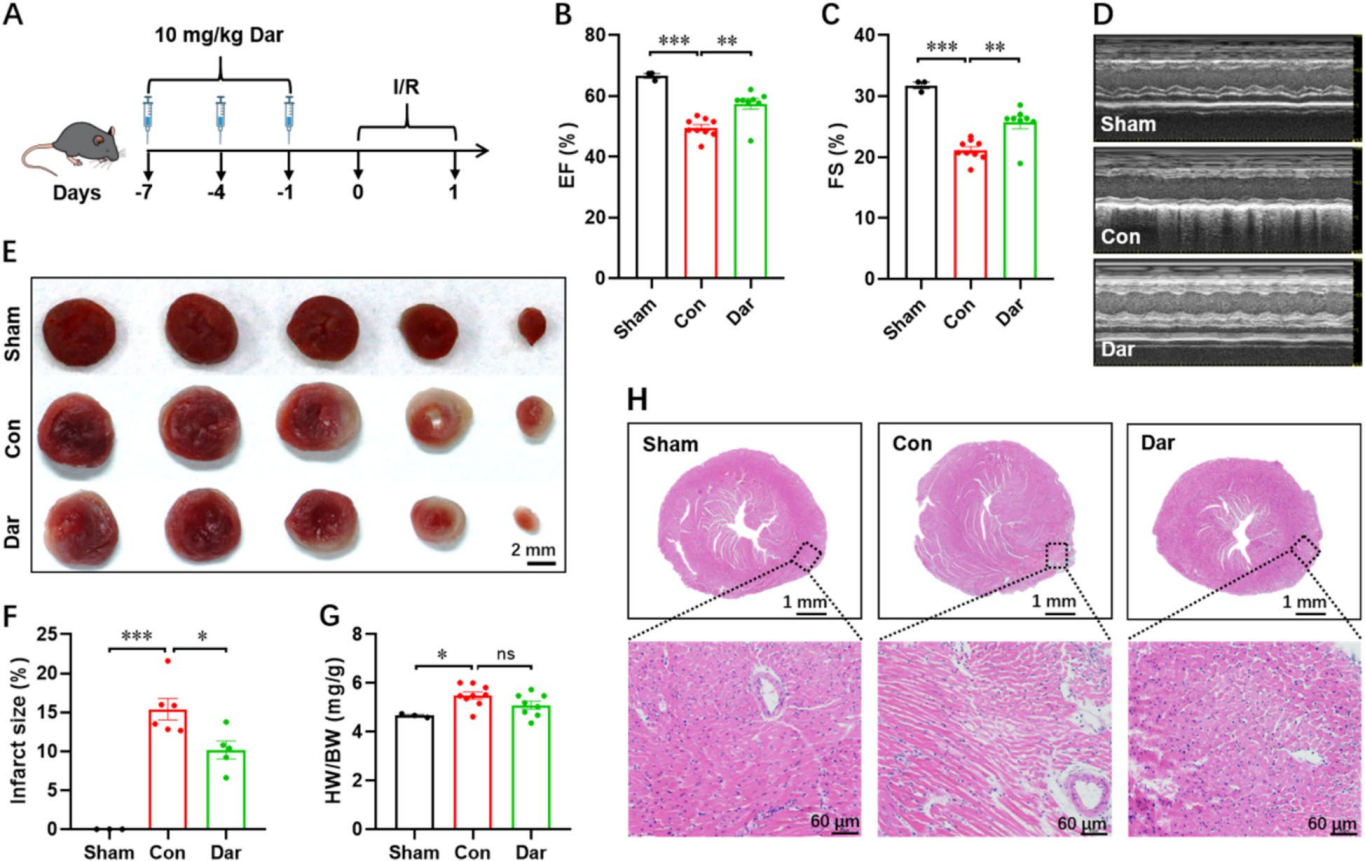
Dar mitigates I/R-induced cardiac injury in mice. **A** Schematic of Dar administration followed by myocardial I/R injury (ischemia for 45 min followed by reperfusion for 24 h). The mice received I/R injury and vehicle injection were used as control group. The mice only received vehicle injection but not I/R injury were used as sham group. **B**–**D** Quantification of LVEF (**B**) and LVFS (**C**) levels as well as representative images of M-model echocardiography (**D**) at 24 h post-I/R injury (*n* = 3 ~ 9 mice). **E** and **F** Representative images of heart TTC staining (**E**) and quantification (**F**) of infarct size at 24 h post-I/R injury (*n* = 3 ~ 6 mice). **G** HW/BW ratio at 24 h post-I/R injury (*n* = 3 ~ 9). **H** H&E staining was used to observe the histopathologic changes of myocardial tissues post-I/R injury. All data are presented as the mean ± SEM of three separate experiments. *p < 0.05, **p < 0.01, ***p < 0.001 versus control. Ns denotes there was no significant difference

To investigate whether Dar administration protects against I/R injury, adult male mice were intraperitoneally injected with Dar (10 mg/kg) every three days for one week prior to I/R injury, followed by ischemia induction for 45 min and reperfusion for 24 h (Fig. 5A). The echocardiography results revealed a notable decline in cardiac function in the control mice compared with the sham group following I/R injury, while Dar pretreatment effectively ameliorated the cardiac function in the hearts upon I/R injury (Fig. 5B–D). In parallel with the enhanced cardiac function, mice subjected to Dar pretreatment had smaller infarct sizes than that in control mice, as shown by TTC staining (Fig. 5E, F). The HW/BW ratio was increased by I/R injury, whereas Dar administration had no significant impacts on the HW/BW ratio in the mice upon I/R injury (Fig. 5G). H&E staining revealed that I/R caused significant cardiac damage, characterized by extensive disruption of myocardial fibers, while the myocardial injury was markedly reduced by the Dar pretreatment (Fig. 5H). Taken together, these data imply that Dar pretreatment might protect the heart from I/R injury.

### Target prediction of Dar against ischemic heart disease (IHD)

**Fig. 6:**
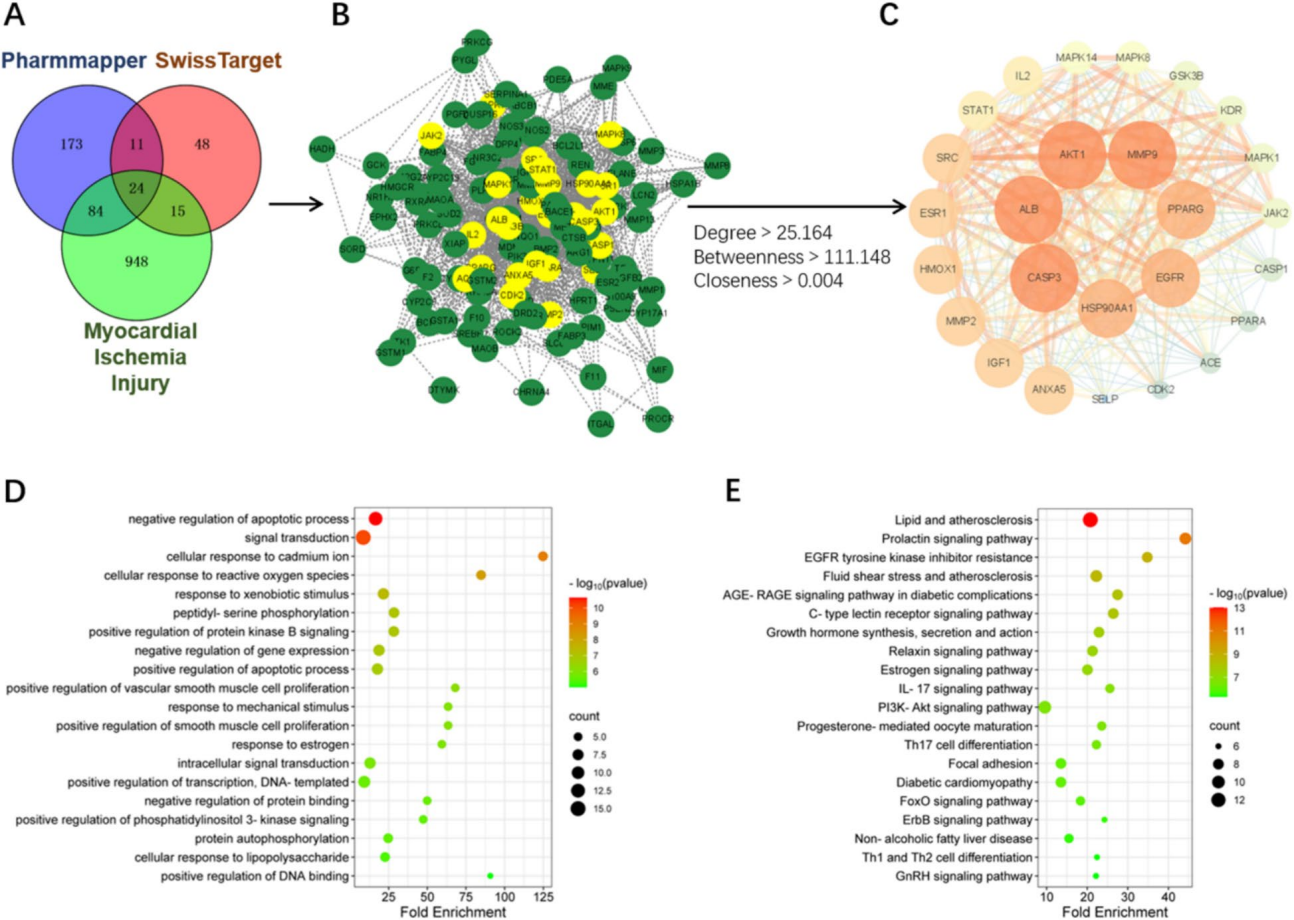
Network pharmacology for predicting the potential targets of Dar against ischemic heart disease. **A** Venn diagram identified 123 overlapping targets between of Dar targets and myocardial ischemic injury targets. **B** The protein–protein-interaction network of the 123 overlapped targets from A. **C** The 26 key targets of Dar were screened by the topological analysis via Cytoscape software. **D** and **E** GO (**D**) and KEGG (**E**) analysis of the screened 26 key targets

To explore the underlying mechanism and targets of Dar in IHD, network pharmacology was utilized in this study. Initially, 390 putative targets of Dar were obtained using the Pharmmapper and SwissTarget databases based on the structure of Dar. Next, 1,071 IHD-related disease targets from four databases including DisGeNET, GeneCards, TTD, and CTD were identified. By overlapping these putative targets of Dar with IHD-related disease targets, 123 intersection targets of Dar against IHD were acquired (Fig. 6A). Then, these intersection targets for Dar treatment of IHD were imported into the String database to construct the PPI protein interaction network (Fig. 6B). Based on the value of degree, betweenness, and closeness, the top 26 key targets including MMP9, AKT1, ALB, CASP3, and HSP90AA1 were selected (Fig. 6C).

**Fig. 7:**
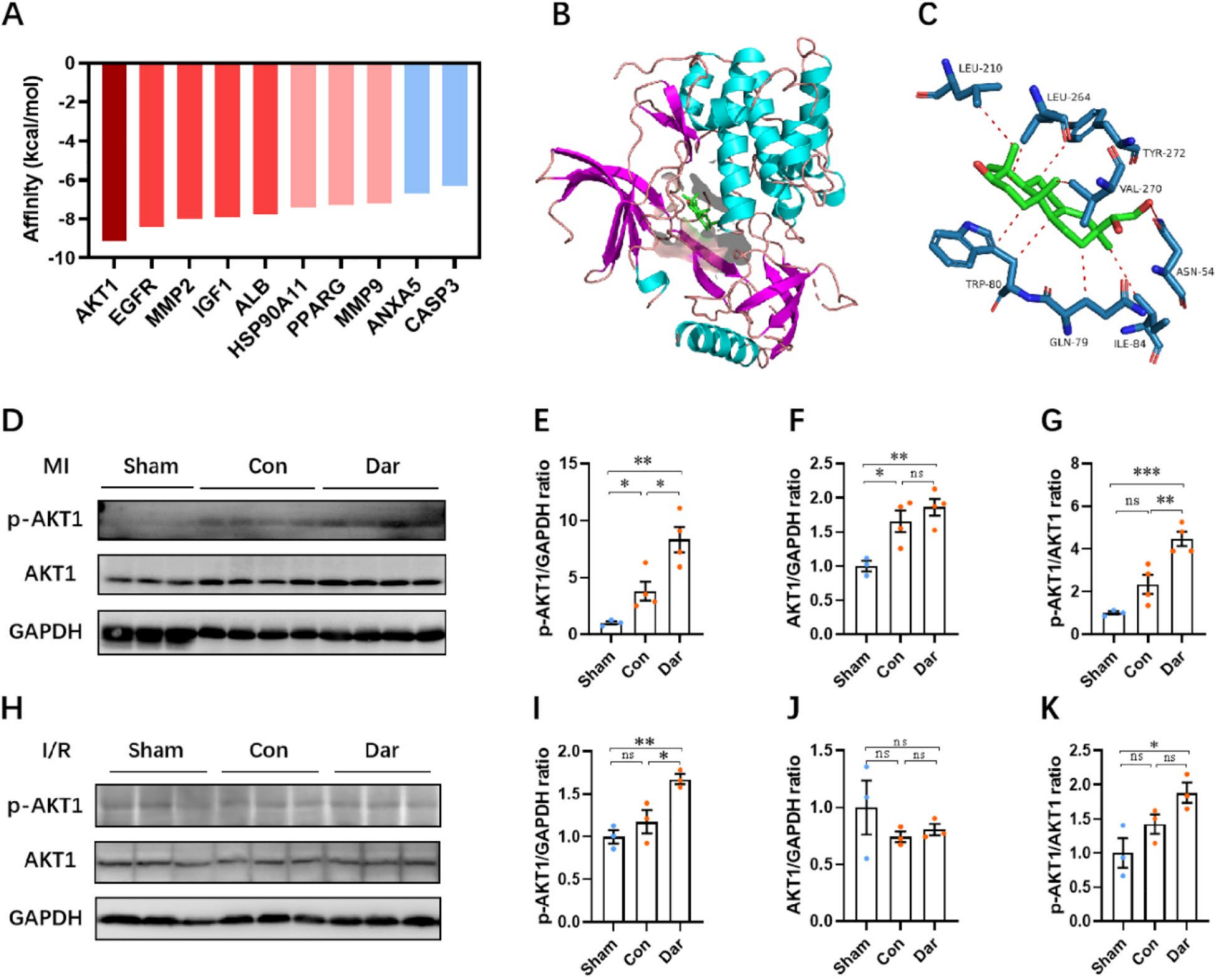
Validation of the target of Dar via molecule docking and western blotting assay. **A** The molecule docking assay was used to predict the binding affinity between Dar and its targets. **B** The best docked pose of Dar within AKT1 (PDB: 3O96). **C** Predicted non-covalent interactions between Dar and AKT1 protein. **D**–**G** Representative images (**D**) and quantification (**E**–**G**) of western blotting for p-AKT1 and AKT1 expression in the infarcted ventricles at 28 dpMI (*n* = 3 ~ 4 mice). **H**–**K** Representative images (**H**) and quantification (**I**–**K**) of western blotting for p-AKT1 and AKT1 expression in the infarcted ventricles at 24 h post I/R (*n* = 3 mice).All data are presented as the mean ± SEM of three separate experiments. *p < 0.05, **p < 0.01, ***p < 0.001 versus control. Ns denotes there was no significant difference

In order to elucidate the potential functions of these core targets, gene ontology (GO) and KEGG pathway analysis were performed via using the David database. According to the GO enrichment analysis, apoptosis regulation, response to ROS, and other biological processes were significant enriched among the top 20 terms (Fig. 6D). The KEGG enrichment analysis revealed that these core targets of Dar were related to several signaling pathways includingPI3K-AKT pathway (Fig. 6E). To further insight into the binding ability of the predicted key targets to Dar, the binding affinity between Dar and the top 10 targets displayed in Fig. 6C was analyzed using the Autodock Vina software respectively. The docking result showed that the binding affinity of Dar to AKT1 protein was −9.1 kcal/mol, which was significantly lower than that of the other targets (Fig. 7A). The docked pose of Dar to AKT1 protein showed that Dar could form a hydrogen bond with the Val-270 and Asn54 on AKT1, as well as a van der Waals interaction with Gln-79, Trp-80, Ile84, Leu-210, Leu-264, and Trp-272 (Fig. 7B, C). These results suggest that Dar may form a strong interaction with AKT1 protein, thereby regulating the PI3K-AKT signaling pathway to protect myocardium from ischemic injury.

**Fig. 8:**
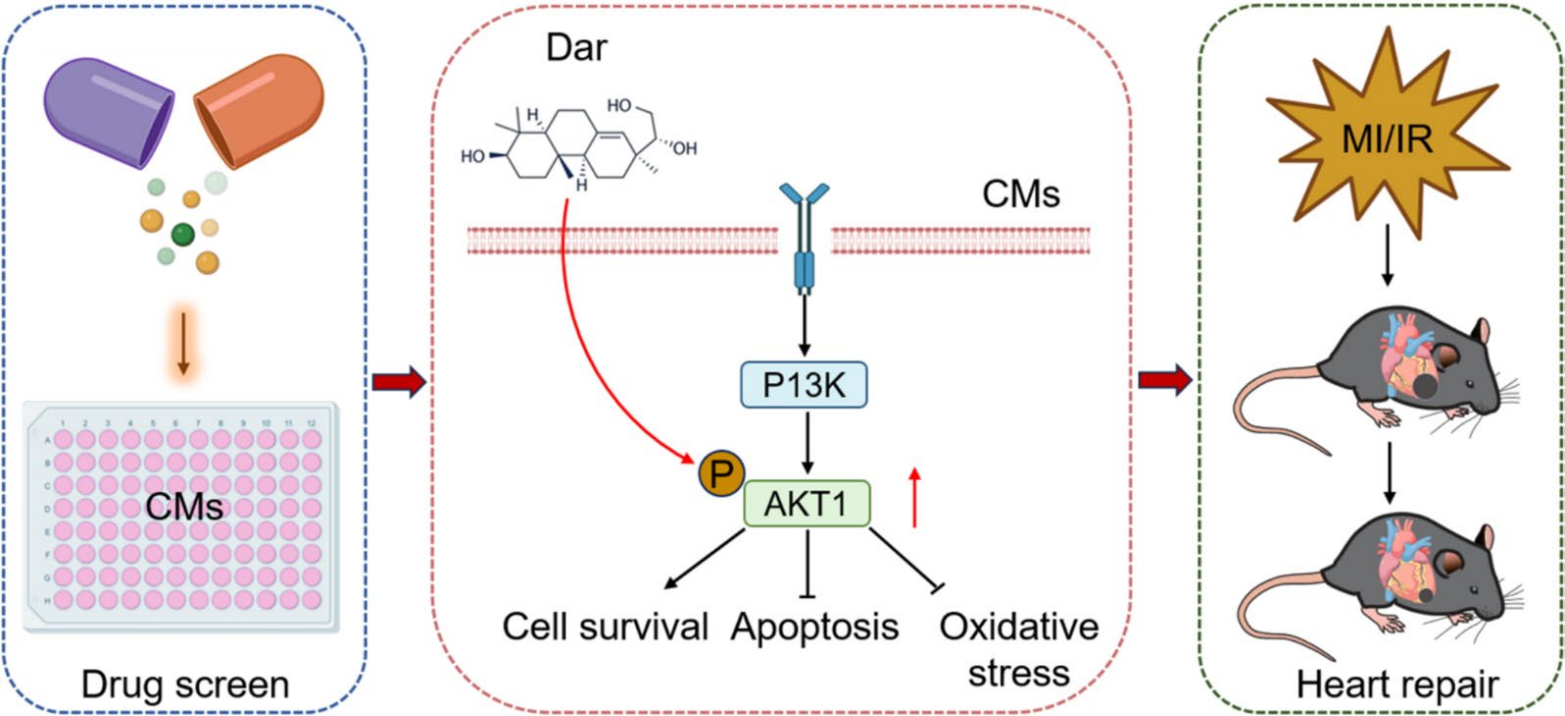
A model of Dar protects heart against cardiac injury. High-throughput screening using CMs identified a small molecule compound named Darutigenol (Dar), which can promote CM survival under ischemic conditions in vitro. Moreover, Dar administration protects adult mice against MI and I/R injury in vivo. Mechanistically, Dar treatment suppresses apoptosis and ROS and promotes survival of CMs upon injury by activating AKT1 pathway. CMs, cardiomyocytes. Dar, darutigenol. MI, myocardial infarction. I/R, ischemia/reperfusion. ROS, reactive oxygen species

To determine the effect of Dar on PI3K-AKT axis, which is important for cardiomyocyte proliferation and heart repair [19, 20], the protein levels of phosphorylated AKT1 (p-AKT1) and total AKT1 in the heart were evaluated using a western blot assay. Our results showed that MI and I/R injury significantly increased the expression levels of p-AKT1 and AKT1 proteins compared with sham group, although the p-AKT1/AKT1 ratio was only modestly upregulated. However, Dar administration further increased the p-AKT1/AKT1 ratio in the hearts upon MI injury (Fig. 7D–K). Taken together, these findings suggest that Dar administration might protect adult mice against MI and I/R injury by promoting the phosphorylation and activation of AKT1 pathway (Fig. 8).

## Discussion

In the present study, we screened a small molecule darutigenol (Dar) from a natural products library, and provided in vitro and in vivo evidence demonstrating the protective function of Dar against myocardial infarction (MI) and ischemic/reperfusion (I/R) injury. We found that Dar treatment can protect primary CMs against ischemic and I/R injury in vitro, by promoting CM proliferation and suppressing CM apoptosis and ROS generation. Moreover, the in vivo protective function of Dar against myocardial infarction (MI) and I/R injury was further evidenced in adult mouse models. Mechanistically, Dar treatment might promote survival of CMs under MI and I/R conditions through the activation of AKT1 pathway, thereby attenuating myocardial ischemic and I/R injury. Our findings suggest that the small molecule Dar is beneficial for myocardial ischemic and I/R injury and provide a new insight into potential therapeutic agents for ischemic heart disease.

In the clinical management of ischemic heart disease, pharmacological treatments have the advantages of lower cost, convenient and controllable administration and interventions [21]. Clinically, anticoagulant and platelet-aggregating drugs, such as aspirin and statins, are frequently employed. Similarly, angiotensin-converting enzyme inhibitors and beta-blockers, which relax blood vessels, lower blood pressure and regulate myocardial oxygen consumption, are commonly used to prevent and relieve myocardial ischemia [22]. Nevertheless, the aforementioned agents are unable to repair the damaged CMs during the period of ischemia, and strategies for treating ischemic myocardium remain a significant challenge. In recent years, researchers have continued to identify the significant roles of small molecules in the treatment of heart disease. These compounds facilitate the repair of cardiac injury by promoting the proliferation of CMs and by reducing inflammatory responses and fibrosis, which in turn enhance heart repair [23–28]. Nevertheless, the process of drug discovery is lengthy and costly, with a relatively low conversion rate from animal to human clinical trials, at less than 8% [29]. Therefore, there is an urgent need to discover new agents for the treatment of ischemic heart disease.

Many previous studies have suggested that enhancing the capacity of CM proliferation is an effective strategy to promote regeneration and repair of the injured heart [30–33]. In this study, a drug screening assay based on the proliferation of CMs identified a natural monomer product Darutigenol (Dar), which could significantly promote neonatal CM proliferation in vitro. Dar is natural monomer product from the herb *Herba Sigesbeckia* (HS). HS is recorded in the Pharmacopoeia of the People's Republic of China as a traditional Chinese medicine for the treatment of rheumatoid arthritis, hypertension, snake venom and malaria. However, little is known about its efficacy in heart repair. The results of our animal experiments demonstrated that Dar could alleviate MI-induced cardiac injury in adult mice by mitigating the development of fibrosis and apoptosis, thereby decreasing the infarcted area and improving cardiac function. However, the administration of Dar failed to promote the proliferation of CMs in the infarcted area in the adult mice, as evidenced by the Ki67 labelling assay. Unlike neonatal CMs, adult CMs belong to terminally differentiated cells which lose the self-renew ability [30, 34]. Promoting the proliferation of adult CMs remains a significant challenge that requires urgent attention. Although Dar was unable to promote adult CM proliferation, our data suggest that Dar administration can enhance heart repair, at least in part, through suppressing fibrosis and apoptosis.

In this study, a network pharmacology study revealed that the predicted targets of Dar against ischemic heart disease were significantly enriched in apoptosis regulation and response to ROS. Apoptosis has been identified as a key factor leading to the deterioration of CMs. The inhibition of CM apoptosis has thus emerged as a promising therapeutic strategy for mitigating heart injury [35, 36]. Furthermore, a KEGG pathway analysis revealed significant enrichment of apoptosis and proliferation related pathways, including the PI3K-AKT signaling pathway and the FoxO signaling pathway. Importantly, Dar and AKT1 protein were shown to have the strongest binding capacity in a molecular docking assay. The PI3K-AKT signaling pathway has been identified as a critical regulator of many biological processes such as survival, proliferation, and apoptosis [37, 38]. Accumulated evidence has demonstrated its involvement in the pathogenesis of various diseases including MI [39]. In response to external or internal stimulations, PI3K kinase converts phosphatidylinositol 4,5-bisphosphate (PIP2) to phosphatidylinositol 3,4,5-trisphosphate (PIP3). Subsequent to this, PIP3 binds to the N-terminal Pleckstrin homology (PH) domain of the AKT protein, resulting in AKT translocating from the cytoplasm to the cell membrane and exposing two amino sites of Ser473 and Thr308. Thereafter, 3-phosphatidylinositol-dependent protein kinase 1 (PDKI) and 3-phosphatidylinositol-dependent protein kinase 2 (PDK2) phosphorylate the above two sites of AKT, thereby leading to the activation of the AKT signaling pathway [40–43]. AKT1, as a core molecule of the PI3K/AKT signaling pathway, has been implicated in ischemic heart disease (IHD) in many previous studies. Previous research has shown that activation of the PI3K/AKT pathway can attenuate ischemic cardiac insult by inhibiting oxidative stress [44, 45], reducing cardiomyocyte apoptosis [46], suppressing the inflammatory response [45, 47], regulating autophagy [48], and promoting cardiomyocyte proliferation [49]. Consistent with the cardioprotective effect of AKT, the result of our Western blot assay showed that Dar administration after MI and I/R insult significantly increased the expression levels of p-AKT1/AKT1 in the heart of adult mice, suggesting that Dar may activate the PI3K-AKT signaling pathway to promote cardiac repair. In line with our results, the important role of AKT1 in tissues repairs has been reported in previous studies. It has been reported that treatment with AKT1 significantly increased wound closure of heart valve interstitial cells (VICs), while AKT1 inhibition reduced wound of VICs compared with nontreated cells [50]. Moreover, AKT1 activation is required for muscle differentiation [51]. In addition, AKT1 activation facilitates cardiac repair in adult mice by activating EGR1 pathway [52]. These previous studies supported, at least in part, our data showing that AKT1 might be the target of Dar in protecting CMs in hearts upon MI and I/R injury.

Recent studies have identified several natural small molecules and traditional Chinese medicine (TCM) formulas with cardioprotective effects. For example, Ginsenoside Rh1 active SIRT3 to suppress oxidative stress and mitochondria damage to protect against myocardial ischemia-induced mitochondrial dysfunction [53]. Ginsenoside Rb2 inhibits p300-mediated SF3A2 acetylation for promoting Fscn1 expression to protect cardiomyocytes from ischemic/reperfusion injury [54]. Bufalin and lycorine alleviate Ang II-induced cardiac remodeling by inhibiting myocardial fibrosis. Kaempferol alleviates myocardial ischemia injury by reducing oxidative stress via the HDAC3-mediated Nrf2 signaling pathway [55]. Yiqi Huoxue (YQHX) prescription attenuates oxidative stress by improving the structure and function of mitochondria to mitigate myocardial ischemia/reperfusion injury [17]. Xinmai’an tablets deduce mitochondrial oxidative stress damage by activating the AMPK/SIRT1/PGC-1α pathway to alleviate myocardial ischemia/reperfusion injury [56]. Mechanistically, reducing the level of oxidative stress and apoptosis after myocardial ischemic injury is an effective strategy to promote myocardial protection against myocardial injury. Our results also demonstrated that Dar mitigates ischemic cardiac injury by reducing the level of ROS and apoptosis, although its molecular mechanisms are different from the above-mentioned drugs. Moreover, the natural product Dar possesses the advantages of low toxicity and ease of access, rendering it a promising candidate for the treatment of ischemic heart disease.

Several limitations of this study warrant consideration. First, our results have demonstrated the cardioprotection effect of Dar on MI- and I/R-induced cardiac insult may be attributed to the activation of AKT pathway. However, the inhibitors of AKT pathway were not administered in vivo in the presence of Dar, which would provide more conclusive evidence regarding the involvement of AKT in mediating the protection. Second, individual variability in Dar's efficacy may arise from host factors such as genetic polymorphisms in drug metabolism pathways [57]. Third, the complexity of the pathological microenvironment has the potential to compromise the efficacy of Dar. Hypoxia and matrix remodeling in areas of advanced myocardial fibrosis can impede drug penetration, thereby reducing its therapeutic effect [58]. Fourth, to further enhance the clinical translational value of Dar, further evaluation is necessary to ascertain whether Dar can elicit cardioprotective effects by modulating the AKT pathway in large animals, such as pigs, and in human myocardial (hiPSC-CMs) and organoid models.

## Conclusion

In conclusion, our screening found that the natural product Dar could significantly promote neonatal mouse cardiomyocyte proliferation and protect cardiomyocytes from ischemic and I/R injury in vitro. Furthermore, Dar was able to ameliorate MI- and I/R-induced cardiac injury in adult mice by reducing cardiac fibrosis and apoptosis. Mechanistically, our data revealed that Dar may protect heart against MI and I/R injury via activating AKT1 pathway. Taken together, our data provide a novel potential candidate for the treatment of ischemic heart disease.

## Data Availability

The authors confirm that the data supporting the findings of this study are available within the article.

## Abbreviations

CMs: Cardiomyocytes
MI: Myocardial infarction
I/R: Ischemia/reperfusion
Dar: Darutigenol
HS: *Herba Sigesbeckia*
DMEM: Dulbecco's modified eagle medium
FBS: Fetal bovine serum
CCK-8: Cell counting kit 8
OD_450nm_: Optical density at 450 nm
SPF: Specific pathogen-free
P3: Postnatal day of 3
EDTA: Ethylenediaminetetraacetic acid
BDM: 2,3-Butanedione-2-monoxime
pH3: Phosphorylated histone H3
PBS: Phosphate-Buffered Saline
DAPI: 4',6-Diamidino-2-phenylindole
LAD: The left anterior descending artery
EF: Ejection fraction
FS: Fractional shortening
WGA: Wheat Germ Agglutinin
TTC: 2,3,5-Triphenyl tetrazolium chloride
TUNEL: TdT-mediated dUTP Nick-End Labeling
IHD: Ischemic heart disease
